# Chiropractic: a bigger family than we might think

**DOI:** 10.1186/s12998-019-0272-9

**Published:** 2019-08-06

**Authors:** Charles Blum

**Affiliations:** Santa Monica, USA

I enjoyed the article “Chiropractic, one big unhappy family: better together or apart?” [1] However I feel the article misses crucial issues in the chiropractic family. For instance the concept that chiropractors utilizing the phrase subluxation are not utilizing evidence-based information is a bit “off the mark.” Having attended the International Research and Philosophy Symposium [2] and read Annals of Subluxation Research [3] it is clear there are chiropractors utilizing the term “subluxation” attempting to perform research and develop an evidence-based rational. Within the technique sacro occipital technique (SOT) there are chiropractors that use the word “subluxation” to describe what they are treating and others that do not. Most SOT chiropractors treat the myofascia, use rehabilitative exercises, adjust extremities, and even use physical therapeutic modalities.

If I were to try to help mediate this chiropractic family dispute I would ask that both sides of the “subluxation” and “evidence-based” chiropractic family attempt to respect one another and try to find commonalities. There seems to be a summary dismissal from both sides of the family of the “other’s” perspective and values. However a larger issue is the division between chiropractors in clinical practice and those primarily in academics [4]. Each has a completely different worldview, perspective, and tend to devalue the other.

There are various factors to consider and here are three examples:

1. Generally chiropractors in clinical practice do not adequately understand the research process and miss issues of coincidence, causality, and causality in the clinical encounter. On the other hand often academicians tend to have a reductionistic perspective, which is not expansive enough to deal with the multifaceted clinical experiences and the various responses of patient subsets to chiropractic interventions [5].
2. Learning preferences tend to characterize perspectives in the chiropractic clinical and academic communities [5, 6]. Clinicians tend to be kinesthetic learners and want to “feel” what they are doing to determine its value. Chiropractic academicians tend to be more visual/auditory learners and determine value through reading and/or writing.
3. Having academicians interested in what clinicians are finding and the clinicians learning to be responsible for their claims would help these two factions move towards greater family harmony.

I believe that the future of the chiropractic family would be rich and diverse if there was a greater openness to learn and share without as much criticism and judgment.

Understanding we all process information differently and have good intentions, could help build a relationship of growth and cooperation.

## Response from the authors

Charlotte Leboeuf-Yde, Stanley Innes, Kenneth Young, Gregory Neil Kawchuk, Jan Hartigsen.

We would like to thank Dr. Blum for taking the time to respond to our article “Chiropractic, one big unhappy family: better together or apart” [1]. Specifically, we would thank Dr. Blum for re-emphasizing the vast diversity of opinion within our profession regarding its future direction. This diversity includes a historic view that the profession can be unified through mutual tolerance. We certainly agree that this is a possibility, but one with low probability. There have been decades of failure to date, a widening gap between traditional and evidence-based practice, and the abandonment of unity as a strategy by our profession’s leading organizations. The argument in Dr. Blum’s letter reinforces the idea that chiropractic is composed of two groups, incompatible in intellectual approach, paradigm of health, and professional focus.
